# Chiral‐Induced Spin‐Polarized Molecular Switching in a Magneto‐Controlled 2D System using Electrical Readouts

**DOI:** 10.1002/smll.202513626

**Published:** 2026-03-15

**Authors:** Yiming Lei, Ángel Campos‐Lendínez, Irena Spasojevic, Xavier Sala, Jordi García‐Antón, Jordi Sort, Jose Muñoz

**Affiliations:** ^1^ Departament de Química Universitat Autònoma de Barcelona Barcelona Spain; ^2^ Departament de Física Universitat Autònoma de Barcelona Barcelona Spain; ^3^ Catalan Institute of Nanoscience and Nanotechnology (ICN2) CSIC and BIST Barcelona Spain; ^4^ Institució Catalana de Recerca i Estudis Avançats (ICREA) Barcelona Spain

**Keywords:** 2D germanene, chiral‐induced spin selectivity, chiral materials, quantum switch, spintronics

## Abstract

Molecular switches that exhibit bistable electron spins under ambient conditions have attracted growing interest due to their potential applications in quantum technologies, enabling exploitation of the chiral‐induced spin selectivity (CISS) phenomenon on electron transfer processes. However, conferring chirality to 2D materials remains a major challenge in Materials Chemistry. Herein, we report the molecular engineering of a chiral spin‐filtering 2D material ―viz. 2D germanane (2D─GeH)―functionalized by covalent anchoring of chiral cysteine molecules via nucleophilic substitution. By interfacing the resulting chiral 2D material with a ferromagnetic electrode, we demonstrate the dynamic control of spin polarization by manipulating the external magnetic field, leading to two well‐defined and electrically distinguishable quantum states. Additionally, the spin polarization direction can be tailored on‐demand via enantiomeric configuration of the chiral ligand, promoting spin‐dependent electron transport. These findings establish a platform for fine‐tuning the spin polarization in chiral 2D materials, offering new opportunities for writing, erasing, and reading unconventional spin‐selective molecular switches, and thereby paving the way for advances in quantum information processing and spintronics applications.

## Graphical abstract

Through molecular engineering, an unconventional chiral 2D platform based on covalently functionalized 2D germanane achieved magneto‐controlled spin‐dependent electron transport, which can be further tuned through the enantiomeric configuration of the chiral ligand. This is the first demonstration of reversible bistable quantum states in a chiral 2D material triggered by the molecularly implanted CISS effect.

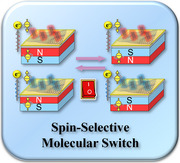

## 1 Introduction

Molecular switches play a crucial role in dynamically controlling the properties of materials and devices ad hoc [1, 2, 3]. The fundamental principle of molecular switches relies on bistability, in which two distinct thermodynamically stable states can be reversibly interconverted by manipulating an external stimulus (*input*) [4, 5]. When these states can be electrically read out (*output*), molecular switches become particularly appealing for digital information processing, since they can serve as fundamental binary logic components (e.g., 0/1, ON/OFF, or YES/NO) in molecular computing [6], memory devices [7], logic gates [8], and/or (bio)sensors [9].

Among the wide library of bistable molecular components, chiral molecules have attracted significant attention owing to their inherent spin‐dependent electron transport properties, a phenomenon known as Chiral‐Induced Spin Selectivity (CISS) [10, 11]. The CISS effect describes the ability of chiral molecules to act as spin filters, selectively transmitting electrons with a preferred spin polarization determined by their enantiomeric form [12, 13]. Moreover, when interfaced with ferromagnetic materials, these chiral‐induced spin‐polarized electronic states can be dynamically controlled by modulating the orientation of an external magnetic field (H) [14]. Consequently, CISS‐based systems are poised to serve as pivotal building blocks for next‐generation quantum technologies, where precise spin manipulation is essential. Despite recent advances, achieving CISS in extended 2D systems, beyond isolated molecules, continues to be one of the central challenges in Materials Chemistry. To date, several chiral molecule‐rich systems have demonstrated electrically detectable CISS responses—commonly via magnetic conductive‐probe atomic force microscopy [15, 16, 17] and/or magnetoresistive device configurations [18, 19]—, displaying H‐dependent spin‐selective transport and reversible switching upon field reversal. While chiral induction has been achieved in some inorganic 2D materials (e.g., graphene, perovskites, metallic microrobots, transition metal carbides, or transition metal chalcogenides) by anchoring chiral molecular selectors [9, 15 20, 21, 22, 23], the interfacial exploration of spin as a state variable for the development of CISS‐driven molecular switches still presents open opportunities. Compared to bulk 3D systems, 2D materials offer confined geometry and enhanced opportunities to engineer spin–orbit coupling (SOC), enabling a more efficient and controllable way to manipulate spin [24]. In particular, the buckled honeycomb structure of 2D germanene (2D─GeH) induces an intrinsic SOC that opens a small band gap at the Dirac point, which can facilitate spin‐polarized transport and electron mobility [25]. These features make 2D─GeH an ideal platform for exploring spin polarization through CISS effect while enabling complementary interfacial electrical readout modes beyond solid‐state conduction.

**SCHEME 1:**
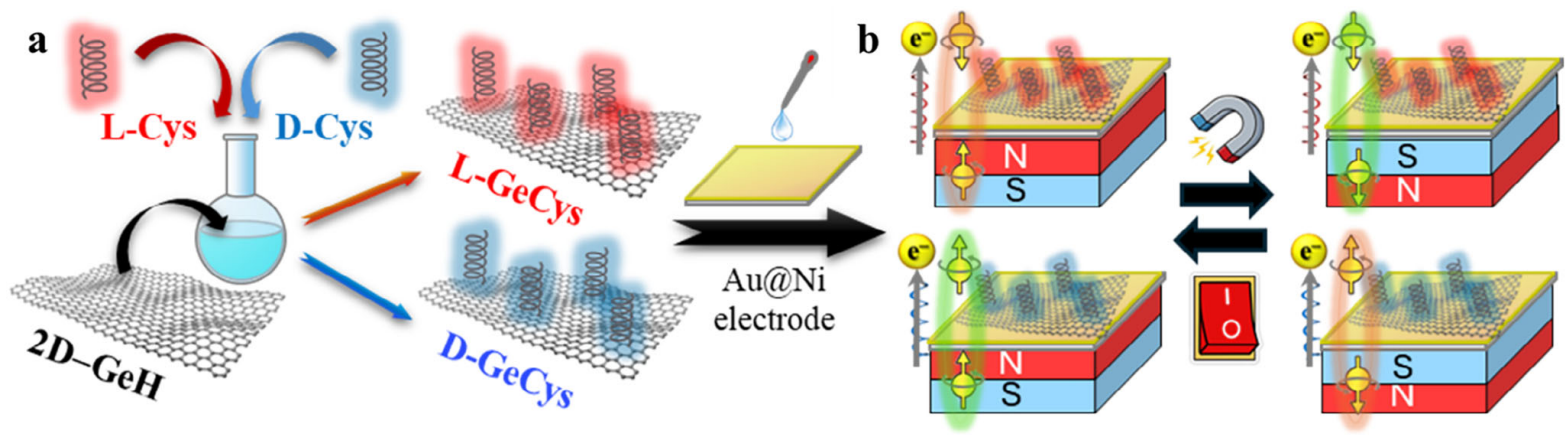
Illustration of the chiral 2D system and the chiral‐induced spin‐polarized molecular switch. (a) Description of the synthetic approach for the development of chiral 2D germanane derivatives (L‐GeCys and D‐GeCys) by covalently anchoring Cys isomers on 2D─GeH via thiolation chemistry. (b) Schematic diagram of the device structure through interfacing the chiral 2D germanene derivatives into a ferromagnetic Au‐coated Ni (Au@Ni) electrode, and the magnetic control of the CISS effect for electrically monitoring an unconventional spin‐selective molecular switch.

Herein, a chiral 2D system with built‐in CISS effect has been molecularly engineered to enable electrical readout of a magnetically driven spin‐polarized molecular switch, where a bistable spin‐polarized switching state emerges from spin‐dependent electron transport within the chiral 2D framework. For this goal, an emerging inorganic 2D material, such as 2D─GeH, has been covalently functionalized with cysteine (Cys) isomers via our recently reported thiolation chemistry approach [26], leading to chiral 2D germanane derivatives―viz. L‐GeCys and D‐GeCys systems (see Scheme 1 for illustration). A set of material characterization techniques confirmed the successful transfer of the inherent chiral features of Cys to 2D─GeH. Then, the resulting chiral 2D systems were interfaced with a ferromagnetic electrode for electrical readout of a spin‐polarized molecular switch triggered by modulating the orientation of an external magnetic field (H), in which two different electrical states can be distinguished using electrochemical impedance spectroscopy (EIS). Remarkably, the electrical output signals of the chiral 2D systems were also tailored by simply substituting the enantiomeric form of the anchored Cys, offering a reliable spin‐encoding mechanism at the molecular level. Finally, Kelvin Probe Force Microscopy (KPFM) measurements were conducted to validate the feasibility of this approach in solid‐state devices, thereby opening up new avenues for the design of next‐generation quantum information processing technologies.

## 2 Results and Discussion

### 2.1 Synthesis and Characterization of Chiral 2D Systems

**FIGURE 1:**
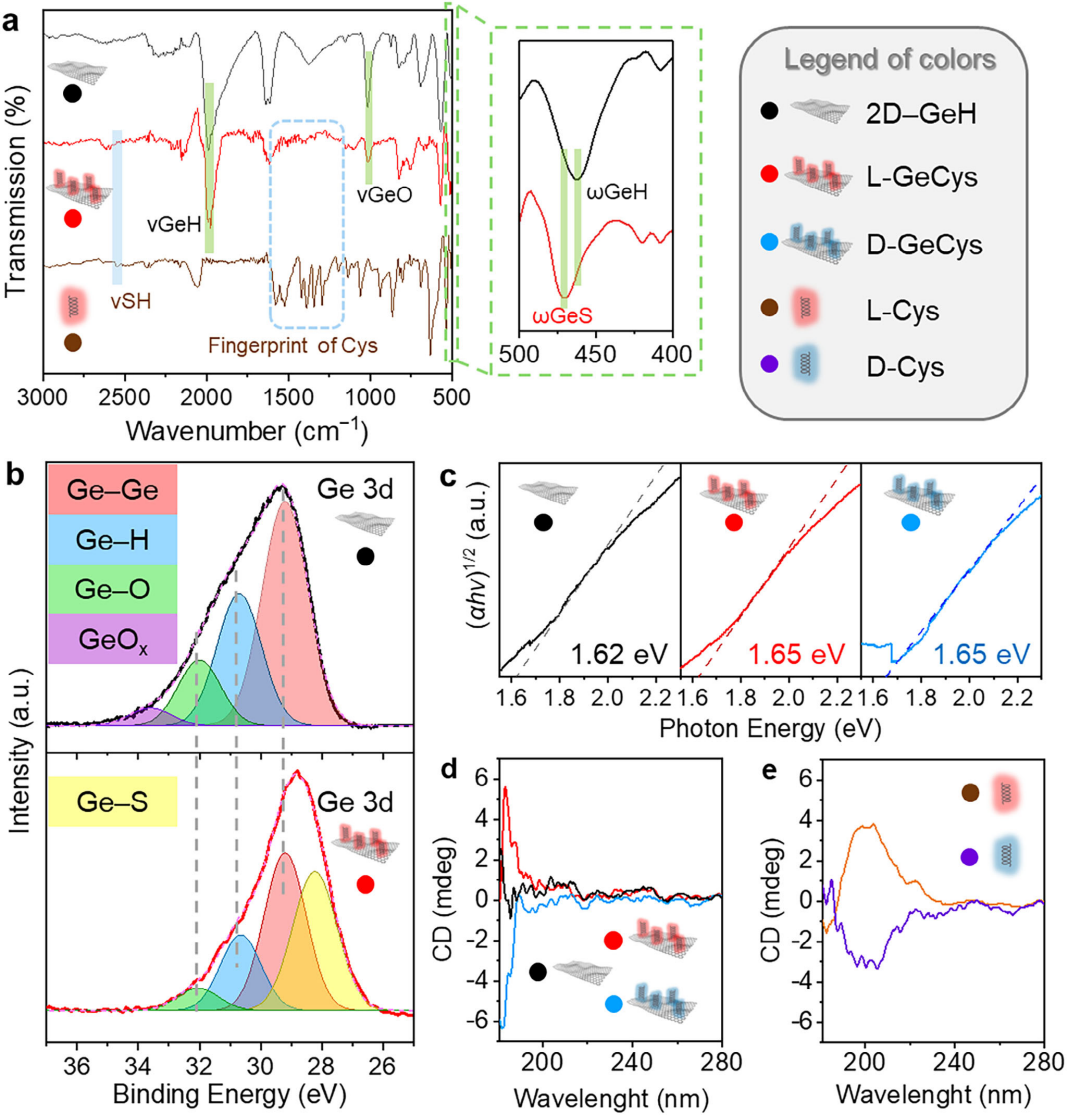
Material characterization of chiral 2D systems. (a) FTIR spectra for pristine 2D─GeH, L‐GeCys and isolated L‐Cys. Inset highlights the new ωGeS contribution. (b) High‐resolution XPS spectra of Ge 3d for pristine 2D─GeH (top) and L‐GeCys (bottom). (c) Tauc plots displaying the optical band gaps for pristine 2D─GeH, L‐GeCys, and D‐GeCys. (d) CD spectra for 2D systems (pristine 2D─GeH, L‐GeCys and D‐GeCys). (e) CD spectra for isolated chiral molecules (L‐Cys and D‐Cys).

Chiral 2D systems were molecularly engineered by covalently anchoring Cys isomers (either L‐Cys or D‐Cys) onto 2D─GeH via Ge─S bond formation, leading to both L‐GeCys and D‐GeCys, respectively. Herein, the H‐terminated ligand of pristine 2D─GeH was substituted by the thiol group (─SH) of Cys through a nucleophilic attack [27]. The successful covalent anchoring of Cys isomers to 2D─GeH was confirmed using multiple characterization techniques (Figure 1).

Figure 1a depicts the Fourier transform infrared spectroscopy (FTIR) spectra of pristine 2D─GeH, functionalized L‐GeCys, and the isolated L‐Cys molecule. Compared to pristine 2D─GeH, the spectrum of L‐GeCys clearly presented new absorption bands between 1600 and 1250 cm^−1^, which correspond to the fingerprint of the isolated L‐Cys. In particular, the bands peaked at 1587, 1393, and 1544 cm^−1^ were assigned to the asymmetric and symmetric COO^–^ stretching modes (υ_a_ C═O and υ_s_C═O) [28, 29, 30], and the N─H bending (δNH) [31, 32, 33], respectively. In addition, the S─H stretching mode (υSH) of the isolated L‐Cys found at 2550 cm^−1^ completely disappeared after material functionalization, suggesting the covalent immobilization via Ge─S bond formation. This result is also in accordance with the blue‐shift observed in the Ge─H wagging mode (*ω*GeH) of pristine 2D─GeH from 462 to 470 cm^−1^ after material functionalization (Figure 1a, inset), which can be assigned to the new Ge─S wagging mode (*ω*GeS) as previously reported [26]. It is also important to point out that the inherent Ge─O stretching mode (υGeO) of pristine 2D─GeH peaked at 987 cm^−1^, generated from the inevitable oxidation under ambient exposure, was notably reduced in L‐GeCys owing to the passivation effect of the thiolation chemistry [34].

The high‐resolution X‐ray photoelectron spectroscopy (XPS) spectra of the Ge 3d orbitals further verify the covalent grafting of L‐Cys onto 2D─GeH (see Figure 1b). While the spectrum of pristine 2D─GeH displayed four typical peaks located at ca. 29.2, 30.7, 32.1, and 33.7 eV, corresponding to Ge─Ge, Ge─H, Ge─O and GeO_x_ contributions [35, 36], the L‐GeCys exhibited a new binding energy peaked at 28.2 eV, which corresponds to the new Ge─S chemical bond [37]. The surface coverage of Cys was estimated from the total area under the curve (Auc) of the Ge─S contribution, yielding an approximate grafting density of 36.7% for L‐GeCys (see Table S1). In line with the FTIR results, the disappearance of the GeO_x_ contribution in the functionalized 2D material must be attributed to the passivation effect of the Ge─S bond formation, demonstrating again the enhanced chemical stability of the molecularly engineered L‐GeCys material.

The optical properties of the 2D systems were explored by means of UV–vis spectroscopy and circular dichroism (CD). Figure 1c presents the Tauc plots derived from UV–vis absorption spectra, illustrating the changes in the optical band gap before (pristine 2D─GeH) and after material functionalization (L‐GeCys and D‐GeCys). Importantly, the optical band gap of pristine 2D─GeH underwent a significant shift from 1.62 to 1.65 eV after the anchoring of Cys isomers, reflecting a consistent tunability in the electronic structure of 2D─GeH due to the covalent immobilization of the chiral molecular components via Ge─S bond formation [27, 38].

Moreover, the chiroptical properties of 2D systems were also investigated, as conferring chirality to 2D materials remains a major challenge in materials chemistry. As shown in Figure 1d, while 2D─GeH did not exhibit chiral signals in the CD spectra, L‐GeCys and D‐GeCys clearly demonstrated mirror‐image chiroptical signals located within the UV range at 180–190 nm, serving as irrefutable proof of their chiroptical activity. Compared to the CD bands of the isolated Cys isomers located at 200 nm (see Figure 1e) [39], this shift also highlights the covalent interaction between the 2D framework and the chiral Cys ligands [40, 41]. Consequently, it is safe to say that the observed CD bands at 180–190 nm originate from the chiral 2D systems rather than from the isolated chiral molecular components, something to point out in the scarce field of chiral 2D materials.

### 2.2 Electrochemical Behavior of Chiral 2D Systems

**FIGURE 2:**
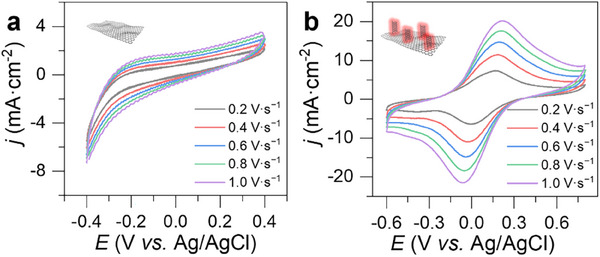
Material characterization via electrochemistry. Cyclic voltammetry of (a) 2D─GeH and (b) L‐GeCys at different scan rates (0.05–1.0 V·s^−1^). Electrochemical measurements were run in a three‐electrode configuration cell filled with 10 mMm PBS at pH 7.2.

Beyond physicochemical characterization, the electrochemical features of the chiral 2D systems were also considered (Figure 2). For this goal, a fixed amount of the chiral 2D systems was drop‐cast onto a fluorine‐doped tin oxide (FTO) substrate, acting as working electrodes. For comparison, electrochemical data of pristine 2D─GeH were also acquired.

On the one hand, the material stability of the cast working electrode was evaluated by means of cyclic voltammetry (CV) in a phosphate‐buffered solution (PBS) at pH 7.2, leveraging the redox responsiveness of the chiral Cys molecule. Figure 2a,b depicts the voltammetric behavior of pristine 2D─GeH and L‐GeCys at different scan rates. While pristine 2D─GeH displayed only non‐Faradaic currents due to its inherent non‐redox activity (Figure 2a), L‐GeCys clearly exhibited a pair of well‐defined redox peaks at 209.5 and −60.3 mV (vs Ag/AgCl), which can be attributed to the Ge─S/Ge─SO_2_H redox couple of the thiol group (Figure 2b) [42, 43]. The low peak‐to‐peak separation (*ΔE*) value obtained (269.8 mV) evidences a high molecular confinement on the 2D material. In addition, an outstanding linear relationship (r^2^) as good as 0.996 was yielded for both anodic and cathodic peaks (Figure S1), indicating a quasi‐reversible surface‐controlled process for the Ge─S/Ge─SO_2_H redox pair [44, 45]. Importantly, the nearly unchanged current intensity signal observed after 50 consecutive cycles (Figure S2) highlights not only the excellent robustness of the chiral 2D system (as expected from a covalently anchored ligand) but also the remarkable stability of the working electrode, with no evidence of material leakage.

On the other hand, and prior to exploiting the CISS effect for monitoring a magneto‐driven spin‐selective molecular switches in the chiral 2D systems via EIS readouts, the EIS signals were recorded by means of charge transfer resistance (*R_CT_*) before and after exposure to a fixed concentration of L‐Amino Acid Oxidase (*L‐AAOx*). For this goal, [Fe(CN)_6_]^3–/4–^ at pH 7.0 was utilized as a benchmark redox marker, since it is known to be very sensitive to small interfacial changes. *L‐AAOx* is a class‐enzyme that selectively catalyzes the irreversible conversion of L‐amino acids (e.g., L‐Cys) into their corresponding α‐keto acids through the following Equation (1):

(1) $$L-\mathrm{amino}\;\mathrm{acid}+O_{2}+H_{2}O\;\overset{L-AAOx}{\to }\;\;\alpha -\mathrm{keto}\;\mathrm{acid}+NH_{3}+H_{2}O_{2}$$

As shown in Figure S3a, the *R_CT_* value of L‐GeCys significantly increased after interaction with *L‐AAOx* due to the selective formation of the α‐keto acid (viz. 3‐mercaptopyruvic acid) [21, 46]. This change can be attributed to the negatively charged form of the α‐keto acid (rather than the zwitterionic form of the anchored L‐Cys at pH 7.0), which promotes a repulsion effect with the negatively charged redox marker (i.e., [Fe(CN)_6_]^3–/4–^), ultimately leading to a decrease in the overall conductivity of the 2D system. In contrast, the *R_CT_* parameter of D‐GeCys remained unaltered after exposure to *L‐AAOx*, demonstrating the enantioselective response of the different chiral 2D systems (Figure S3b). In addition, a control experiment with pristine 2D─GeH was also conducted (see Figure S3c). As expected, pristine 2D─GeH did not exhibit any EIS alteration after exposure to *L‐AAOx* owing to the absence of the chiral molecular component. Consequently, this electrochemical experiment proves the enantioselective behavior of the devised chiral 2D systems.

### 2.3 Monitoring of Quantum States using Electrical Readouts

#### 2.3.1 Electrochemical Monitoring of a Magneto‐Driven Spin‐Polarized Molecular Switch

After successfully demonstrating the synthesis, characterization, and electrochemical performance of the chiral 2D systems, the next step focused on exploring the implanted CISS effect for writing, erasing, and reading an unconventional spin‐polarized molecular switch. For this goal, the chiral 2D systems were drop‐cast onto a Ni electrode sputtered with an Au layer (Au@Ni). While the Ni electrode provides ferromagnetic features, the Au overlayer protects Ni from oxidation [47]. By placing an external magnet beneath the casted working electrodes, the EIS signals of the chiral 2D systems were recorded in PBS buffer at pH 7.0 to detect spin‐dependent charge transfer. Since the redox reaction of the anchored Cys involves electron transport, the spin‐selective nature of the chiral 2D systems shows a clear electrochemical dependence on the direction of H, thereby offering a straightforward way to monitor the CISS effect.

**FIGURE 3:**
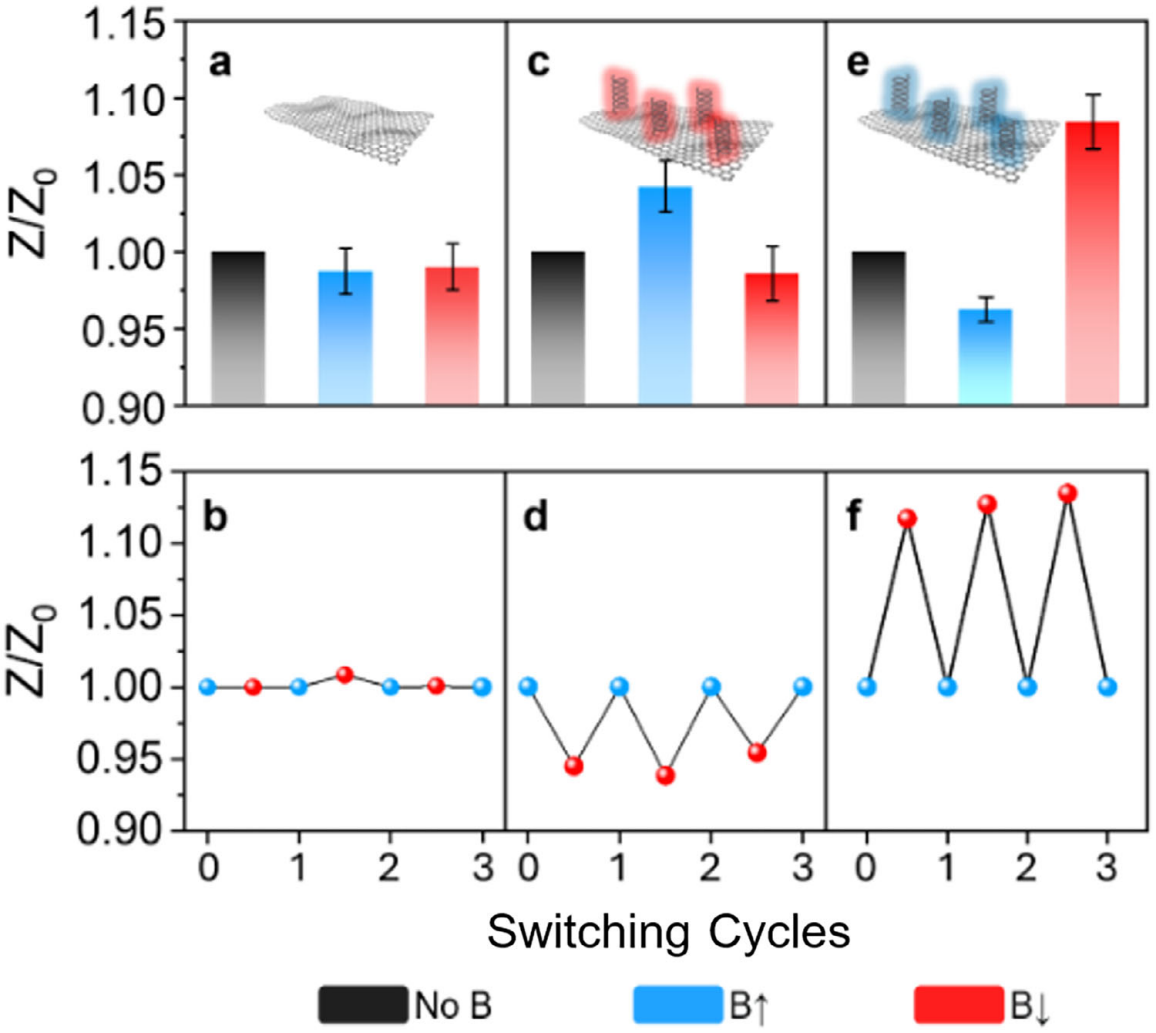
Influence of CISS effect on electron‐spin behavior. Changes in impedance modulus ratio (viz. *Z/Z_0_*) and cyclability of impedance modulus switch as a function of the magnetic field direction achieved for (a,b) 2D─GeH, (c,d) L─‐GeCys, and (e,f) D─‐GeCys. Electrolyte: 10 mM PBS buffered solution (pH 7.2). Voltage: 0.1 V; Frequency: 10 kHz to 0.1 Hz.

The histograms in Figure 3 illustrate the EIS signals as Bode plots (Log *Z* vs Log frequency; see Figure S4) obtained for pristine 2D─GeH (control experiment), L‐GeCys, and D‐GeCys before and after magnetization with both north (H↑) and south (H↓) poles. The reproducibility of the EIS measurements was assessed by testing three different working electrodes (*n* = 3). EIS output signals were represented in terms of the impedance modulus ratio (viz. *Z/Z_0_*), where *Z_0_* and *Z* correspond to the impedance modulus in the absence and presence of the external H, respectively. The higher the *Z/Z_0_* ratio, the better the electron transfer.

As depicted in Figure 3a, the electrochemical performance of pristine 2D─GeH remained unaltered after exposure to the external magnet, independently of the H direction. This trend persisted across several switchable cycles (Figure 3b), indicating that CISS effect cannot occur without the presence of a chiral molecular component. In contrast, the EIS signals of the chiral 2D systems exhibited a clear H‐dependent response due to the transition from low to high spin when coupling the electron spin of the ferromagnetic Au@Ni electrode (see Figure 3c–f).

On the one hand, the *Z* value of L‐GeCys significantly increased with H pointing the north orientation (H↑), while it decreased when the field was flipped south (H↓) (see Figure 3c). This H‐direction‐dependent behavior was further validated through three consecutive polarization cycles (Figure 3d). On the other hand, the chiral counterpart (D‐GeCys) displayed the opposite response (Figure 3e), with *Z* values decreasing under H↑ and increasing under H↓. Moreover, similar EIS signals were recorded over three consecutive switchable cycles (Figure 3f), demonstrating once again the excellent reversibility and robustness of the chiral 2D systems. It is important to note that the observed variation in *Z/Z_0_* ratios between the two chiral 2D systems can be ascribed to differences in functionalization degree, as well as drop‐casting coverage. Nevertheless, this mirror‐like electrochemical dependence, which is dictated by the handedness of the chiral 2D system, serves as irrefutable evidence of the implanted CISS effect [11, 48]. To further validate the CISS effect as the origin of the EIS switching behavior, an additional control experiment was performed using an achiral 2D system (a‐GeCys), which was prepared by covalently functionalizing 2D─GeH with cysteamine (a‐Cys, the achiral counterpart of Cys isomers). Remarkably, a‐GeCys displayed no significant EIS variations upon reversing the H orientation (see Figure S5), confirming that chirality is a *sine qua non* condition for spin‐selective transport. These results underscore the pivotal role of the CISS effect in governing the magneto‐electrochemical behavior of chiral 2D materials.

The crucial contribution of 2D Xene was validated by comparing the spin polarization percentage [%P_EIS_ = (*Z_H↑_–Z_H↓_*) / (*Z_H↑_* + *Z_H_*_↓_) · 100] of L‐GeCys with that of isolated L‐Cys, under identical experimental conditions. For this goal, L‐Cys was cast onto the surface of the Au@Ni electrode, and EIS data were collected before and after magnetization. As shown in Figure S6, the %P_EIS_ of L‐GeCys reached 2.8%, whereas the isolated L‐Cys exhibited a significantly lower %P_EIS_ of 1.9%. This remarkable result demonstrates that the immobilization of chiral molecules onto 2D─GeH can effectively amplify the CISS effect by 68%.

Finally, the switching stability of the chiral 2D system under ambient conditions was evaluated through two complementary tests. On the one hand, multiple consecutive cycles were performed under alternating H orientations. As shown in Figure S7, L‐GeCys maintained consistent switching behavior for 8 consecutive cycles. Beyond this point, the response declined significantly due to material leakage from the Au@Ni electrode surface. On the other hand, short‐term stability of L‐GeCys was assessed by comparing EIS responses at day 1 and day 7 (Figure S8). Importantly, no significant variations were observed, with a %PEIS value of 2.4%, well within the error range of fresh L‐GeCys (2.8% ± 0.4%). This demonstrates that the chiral 2D system retains its functionality over time.

#### 2.3.2 Probing Surface Potential Modulation Induced by Spin Transfer Charge

**FIGURE 4:**
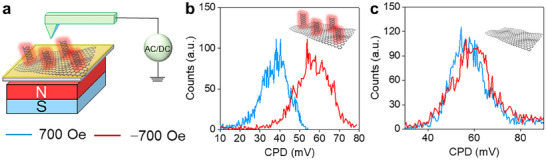
Kelvin Probe Force Microscopy (KPFM) measurements in the presence of the external magnetic field. (a) Illustration of the KPFM experimental setup. An AFM conducting tip was used to image the surface potential of chiral 2D system in the presence of the externally controlled magnetic field. CPD distributions of (b) L‐GeCys and (c) pristine 2D─GeH under two different H orientations (+700/−700 Oe).

Having established the interfacial electron transport characteristics of the chiral 2D systems via EIS, the next step focused on investigating the CISS‐induced surface potential. To this end, Kelvin probe force microscopy (KPFM) was employed to probe in‐solid spin‐dependent surface potential shifts by varying the orientation of H (i.e., ±700 Oe). Figure 4a illustrates the setup utilized for KPFM measurements, in which the ferromagnetic Au@Ni electrode was used to inject spin‐polarized charge carriers into the chiral 2D layer.

As shown in Figure 4b, the L‐GeCys system exhibited a clear dependence of the contact potential difference (CPD) on the orientation of H, resulting in a shift of around 20 mV, consistent with the CPD variations observed for SAMs on Au@Ni electrode.^14^ This shift results in a %P_KPFM_ [%P_KPFM_ = (*CPD_+700_–CPD_–700_*) / (*CPD_+700_* + *CPD*_–700_) · 100] of 20.8%. This dependence is attributed to the CISS effect, which induces spin‐selective interactions at the interface between the Au@Ni electrode and the 2D chiral system (see Figure S9). These interactions lead to a spin‐dependent redistribution of interfacial charge, resulting in a change in the interfacial dipole and a corresponding shift in the vacuum level, as reflected in the measured CPD [14]. Since the magnetization of the Au@Ni electrode lies in‐plane, the relatively small CPD shifts are likely due to the limited magnetic field strength available in the setup, which may not be sufficient to fully reverse the magnetization along the hard (i.e., out‐of‐plane) magnetic axis. Conversely, as shown in Figure 4c, the CPD of the achiral 2D─GeH counterpart displayed negligible CPD variation under the same conditions (Figure S10). This result confirms the key role of chirality in enabling the observed surface potential modulation, and thereby provides compelling evidence of magnetically controlled spin transfer across chiral 2D surfaces [10, 49]. These observations, together with the absence of magnetic or chiroptical response in pristine 2D─GeH, provide strong evidence that the spin chirality is not confined only to the molecular ligands. Instead, it is transferred to the 2D Xene through the covalent Ge─S bonding, resulting in an integrated chiral 2D spin filter in which the CISS effect arises from the entire chiral 2D system rather than from isolated chiral molecules. This distinction is crucial for validating the material's potential in scalable quantum and spintronic applications.

## 3 Conclusion

In this work, we successfully engineered unprecedented chiral 2D─GeH derivatives through covalently anchoring a thiolated chiral precursor (viz. Cys) via nucleophilic substitution. State‐of‐the‐art characterization techniques not only demonstrate the nature of the covalent Ge─S bonding, but most importantly the integration of the inherent chiral features of the molecular precursor onto the 2D material. After casting the chiral 2D systems onto a ferromagnetic electrode, we demonstrated that electron transport is spin‐dependent, making it possible to electrically monitor a spin‐polarized molecular switch at room temperature by simply manipulating the external magnetic field. Further, electron transport can also be customized by tailoring the anchored Cys isomer. The underlying microscopic mechanism involves that i) the SOC between 2D─GeH and chiral molecules lead to an integral chiral 2D system instead of isolated chiral molecules, while ii) the implanted CISS effect from Cys enantiomers results in a mirror image behavior. Consequently, the SOC between 2D─GeH and the covalently bound chiral molecules results in the emergence of a fully integrated, chiral 2D system, where spin chirality is extended across the inorganic 2D framework.

Overall, 2D─GeH has shown to be a promising 2D platform to harbor chiral molecular components for manipulating electron transport at will via CISS phenomenon, thereby paving the way toward the development of novel quantum‐information technologies. Finally, the chemical thiolation method is general and can be readily adapted to alternative 2D materials (e.g., MoS_2_ via vacancy engineering) and/or chiral selectors for task‐specific spintronic applications.

## 4 Experimental Section

### 4.1 Synthesis of Chiral GeCys Systems

The synthesis of chiral 2D systems was carried out via thiolation chemistry (Scheme 1a). First, chiral Cys isomers were treated with HCl to improve solubility and dissolved into a mixed solution containing 4 mL H_2_O and 5 mL acetonitrile (ACN). Then, 2D─GeH was suspended in Ar‐saturated ACN and mixed with 2.5 mM Cys deoxygenated aqueous solution. The mixture was stirred for 12 h to obtain chiral 2D materials (denoted as L‐GeCys and D‐GeCys). The same methodology was used to prepare the achiral 2D system (a‐GeCys), using cysteamine (a‐Cys) as the achiral counterpart of Cys isomers.

### 4.2 Electrode Preparation

150 µL of a 1 mg·mL^−1^ dispersion of samples was drop‐casted onto a ferromagnetic Au‐coated Ni (Au@Ni) working electrode, and a permanent magnet (H) underneath the modified working electrode for monitoring the spin‐selective molecular switch.

### 4.3 Electrochemical Monitoring of CISS Effect

EIS signals were obtained in a standard three‐electrode system involving an Au@Ni, a Pt wire, and an Ag/AgCl (sat. KCl) as the working, counter, and reference electrodes, respectively. The electrochemical cell was filled with a 10 mM PBS buffered solution (pH = 7). With different polarization directions of the permanent magnet, the EIS signal variations were in‐situ monitored according to impedance modulus (*Z*) before and after magnetization with both north (H↑) and south (H↓) poles. In order to test the switching‐ability of chiral 2D systems, the direction of the magnetic field was periodically changed for every 5 min.

### 4.4 Atomic Force Microscopy Setup

AFM imaging was performed on MFP‐3D AFM (Asylum Research). In all the experiments, Ptlr_5_ coated PPP‐EFM tips (Nanosensors) with a stiffness constant k = 2.8 N·m^−1^ were used. KPFM measurements were performed to investigate the electronic structure of pristine 2D─GeH and L‐GeCys as achiral and chiral 2D systems, respectively. A two‐pass scanning mode was used: in the first pass, topographic data were acquired, while in the second pass, a DC voltage was applied to the tip to nullify the electrostatic force between the tip and the sample by compensating for their potential difference. The resulting DC voltage corresponds to the contact potential difference (CPD) between the tip and the sample.

## Conflicts of Interest

The authors declare no conflicts of interest.

## Supporting information




**Supporting File**: smll73048‐sup‐0001‐SuppMat.docx.

## Data Availability

The data that support the findings of this study are available from the corresponding author upon reasonable request.
