# Supplementary material for: Chiral‐Induced Spin‐Polarized Molecular Switching in a Magneto‐Controlled 2D System using Electrical Readouts
Source: Small. 2026 Mar 15;22(25):e13626. doi: 10.1002/smll.202513626 (PMC13137230; doi:10.1002/smll.202513626)
Supplement: Supplementary file 1 — Supporting File: smll73048‐sup‐0001‐SuppMat.docx. [file SMLL-22-e13626-s001.docx]

**Chiral-Induced Spin-Polarized Molecular Switching in a Magneto-Controlled 2D System using Electrical Readouts**

Yiming Lei,^[a]^ Ángel Campos-Lendínez,^[a]^ Irena Spasojević,*^[b]^ Xavier Sala,^[a]^ Jordi García-Antón,^[a]^ Jordi Sort,^[b,c,d]^ Jose Muñoz*^[a]^

^[a] Departament de Química, Universitat Autònoma de Barcelona, Cerdanyola del Vallès, Barcelona, 08193, Spain
[b] Departament de Física, Universitat Autònoma de Barcelona, Cerdanyola del Vallès, Barcelona, 08193, Spain
[c] Catalan Institute of Nanoscience and Nanotechnology (ICN2), CSIC and BIST, 08193 Barcelona, Spain^

^[d] Institució Catalana de Recerca i Estudis Avançats (ICREA), Pg. Lluís Companys 23, 08010 Barcelona, Spain^

**Supporting Information**

**Experimental Section**

| **Chemicals and reagents** **................................................................................................** | **S2** |
| --- | --- |
| **Instrumentation ……………............................................................................................** | **S2** |
| **Chiral activity characterization** **…..................................................................................** | **S2** |
| **Electrochemical monitoring of CISS effect ..................................................................** | **S3** |
| **Atomic force microscopy measurements ....................................................................** | **S3** |

**Supporting Figures**

| **Fig. S1.** Linear dependence of peak current on scan rate for L-GeCys **..............................** | **S5** |
| --- | --- |
| **Fig. S2.** 50 cycles of cyclic voltammograms over L-GeCys **...…......................................** | **S5** |
| **Fig. S3.** Electrochemical impedance spectroscopy with L-AAO **……………......................** | **S6** |
| **Fig. S4.** Bode plots of Log *Z* *vs.* Log Frequency **…..........................................................** | **S6** |
| **Fig. S5.** Control experiment for a-GeCys **.........................................................................** | **S7** |
| **Fig. S6.** Bode plots, impedance modulus ratio, and cyclability for isolated L-Cys **...........** | **S7** |
| **Fig. S7.** Multi-cycle stability test for L-GeCys **..................................................................** | **S8** |
| **Fig. S8.** Short-term stability test for L-GeCys **..................................................................** | **S8** |
| **Fig. S9.** KPFM images and surface potentials of L-GeCys **..............................................** | **S9** |
| **Fig. S10.** KPFM images and surface potentials of 2D–GeH **............................................** | **S9** |

**Supporting Table**

| **Table S1.** Auc and chemical bond contributions of L-GeCys **...........................................** | **S10** |
| --- | --- |

**Experimental Section**

**Chemicals and reagents**

Germanane (2D–GeH), L-cysteine (L-Cys), D-cysteine (D-Cys), cysteamine (a-Cys), and acetonitrile (ACN) were purchased from Sigma-Aldrich (St. Louis, MO, USA). Deionized water from a Milli-Q system (Millipore, Billerica, MA, USA) was used throughout all the experimental parts. All the reagents were directly used as received.

**Instrumentation**

The morphology of as-prepared samples was observed by Transmission Electron Microscopy (TEM, JEM-2011, acceleration voltage of 200 kV). Fourier transform infrared spectroscopy (FTIR) spectra were obtained from a Bruker spectrophotometer Alpha II model with a single reflection diamond attenuated total reflectance (ATR) module. The elemental states were studied by X-ray photoelectron spectroscopy (XPS, SPECS GmbH, Berlin, Germany), using a Phoibos 150 analyzer under the ultrahigh vacuum condition (base pressure 5·10^−10^ mbar) and monochromatic aluminum K_α_ X-ray source (1486.74 eV). UV-vis analysis was performed via a V-730 JASCO spectrophotometer. Circular Dichroism spectra were obtained from Circular Dichroism Spectrometer (JASCO J-815). Electrochemical experiments were conducted in a three-electrode configuration cell (Counter electrode: Pt, reference electrode: Ag/AgCl sat. KCl) by a homemade 3D-printed cell using a PalmSens4 potentiostat/galvanostat equipped with PSTrace software.

**Chiral activity characterization**

The electrochemical discrimination function was confirmed by using L-amino-acid oxidase (*L-AAOx*) as the target detection molecular. The samples were drop-casting onto a FTO substrate. The electrochemical discrimination measurement was conducted in 0.1 M KCl solution containing 10 mM [Fe(CN)_6_]^3−/4–^ as a redox marker. The electrochemical impedance spectroscopy (EIS) signals were collected to monitor the charge transfer behavior variation before and after the addition of *L-AAOx* (50 ng mL^−1^) into the electrochemical electrolyte.

**Electrochemical monitoring of CISS effect**

The electrochemical assay of the CISS effect was conducted by using a ferromagnetic Au-coated Ni (Au/Ni) working electrode and a permanent magnet (H) underneath the modified working electrode. The EIS signals were obtained in a standard three-electrode system involving an Au@Ni, a Pt wire, and an Ag/AgCl (sat. KCl) as the working, counter, and reference electrodes, respectively, with a 10 mM PBS buffered solution (pH = 7). With different polarization directions of the permanent magnet, the electrochemical signal variations were in-situ monitored according to impedance modulus (*Z*). All measurements were run per triplicate (*n=3*). A normalization procedure in terms of impedance modulus ratio (*Z/Z_0_*, where *Z_0_* and *Z* are the impedance modulus values before and after magnetization with both north (H↑) and south (H↓) poles, respectively) was necessary to properly compare the three independent measurement cycles. In order to test the switching-ability of GeCys systems, the direction of the magnetic field was periodically changed for every 5 min.

**Atomic force microscopy measurements**

AFM imaging was performed on MFP-3D AFM (Asylum Research). In all the experiments, Ptlr_5_ coated PPP-EFM tips (Nanosensors) with a stiffness constant k = 2.8 N·m^-1^ were used. KPFM measurements were performed to investigate the electronic structure of pristine 2D–GeH and L-GeCys as achiral and chiral 2D systems, respectively. A two-pass scanning mode was used: in the first pass, topographic data were acquired, while in the second pass, a DC voltage was applied to the tip to nullify the electrostatic force between the tip and the sample by compensating for their potential difference. The resulting DC voltage corresponds to the contact potential difference (CPD) between the tip and the sample. During KPFM measurements, the sample was mounted on a Variable Field Module (VFM) stage, enabling in-situ application of an out-of-plane magnetic field. It is important to point out that the magnetic field applied during KPFM measurements (±700 Oe) corresponds to the maximum achievable with our setup and does not reach saturation magnetization of the ferromagnetic electrode. Thus, the observed CPD modulation is not expected to represent full saturation, and higher fields would likely enhance the effect.

**Supporting Figures**

**
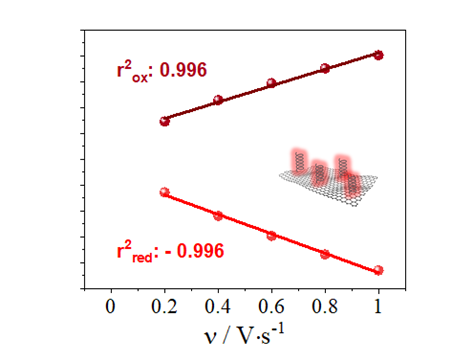
**

**Fig. S1.** Functional relationship between peak current and scan rate according to the CV data of L–GeCys. Electrochemical measurements were run in a three-electrode configuration cell filled with 10 mM PBS at pH 7.2.


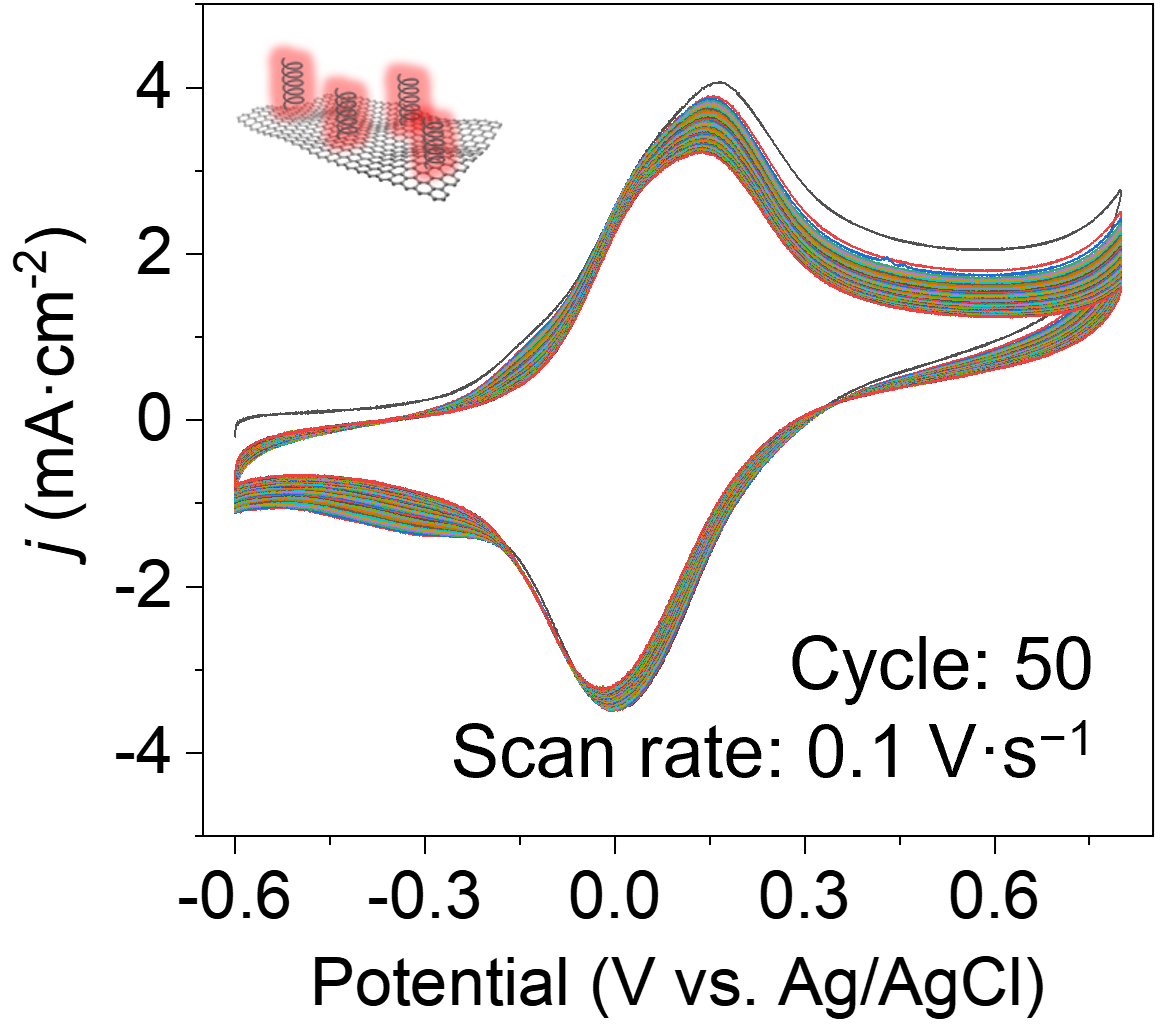


**Fig. S2.** 50 cycles of cyclic voltammograms over L-GeCys. **Reaction Conditions:** Sample drop-casted Fluorine-doped Tin Oxide glass (FTO) as a working electrode, a Pt wire as a counter electrode, and an Ag/AgCl (sat. KCl) as a reference electrode; 10 mM PBS buffered solution (pH = 7).


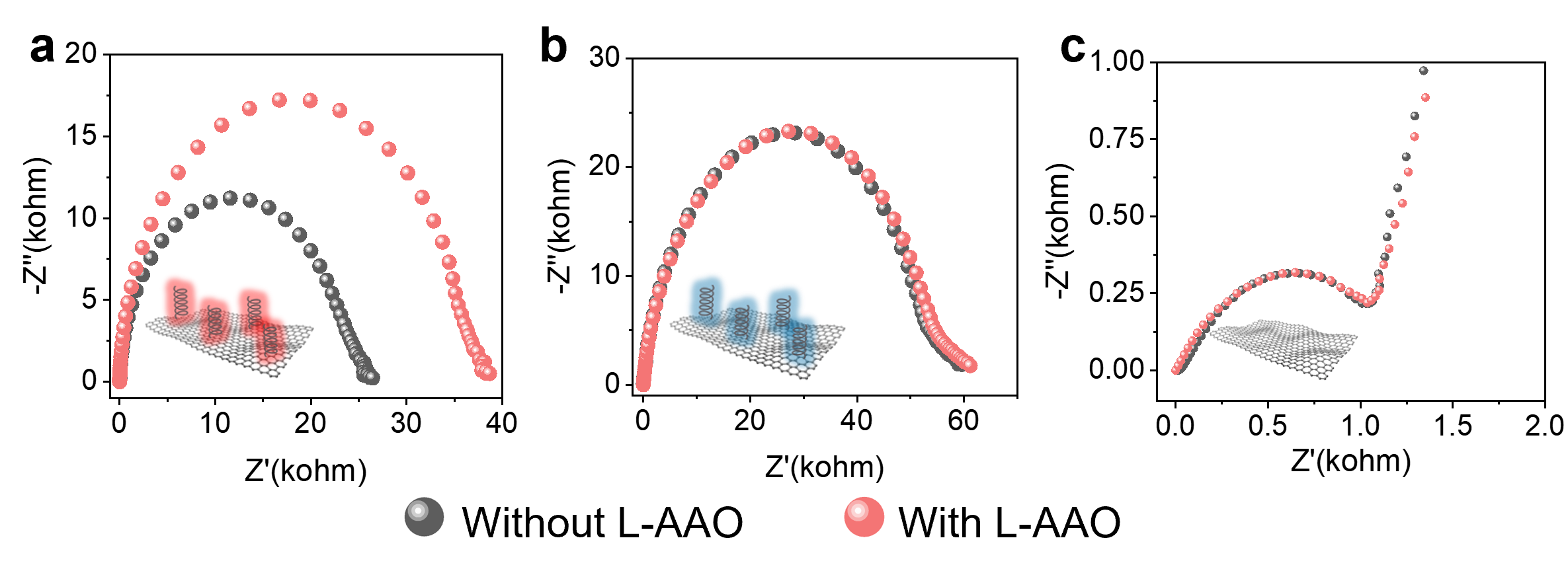


**Fig. S3.** Electrochemical impedance spectroscopy of **(a)** L-GeCys, **(b)** D-GeCys and **(c)** 2D–GeH with or without 50 ng mL^−1^ of L-AAO. Reaction Conditions: Sample drop-casted 3D printing working electrode (carbon + PLA), a Pt wire as a counter electrode, and an Ag/AgCl (sat. KCl) as reference electrode; Electrolyte: 10mM [Fe(CN)_6_]^3−/4−^ and 0.1 M KCl (pH = 7.25); Voltage: 0.25 V; Frequency: 10000HZ to 0.1 HZ.


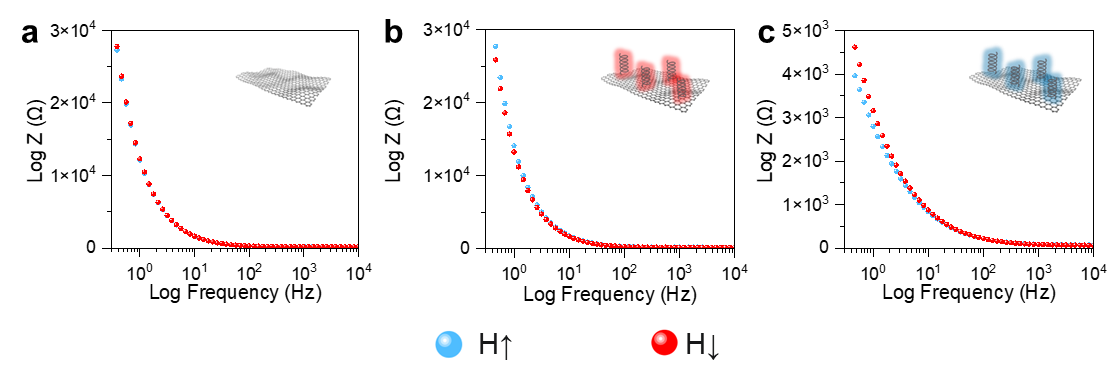


**Fig. S4.** Bode plots of Log Z *vs.* Log Frequency (Hz) for **(a)** 2D–GeH **(b)** L-GeCys, **c** D-GeCys, showing the changes in *Z* after magnetization with both north (H↑) and south (H↓) poles.


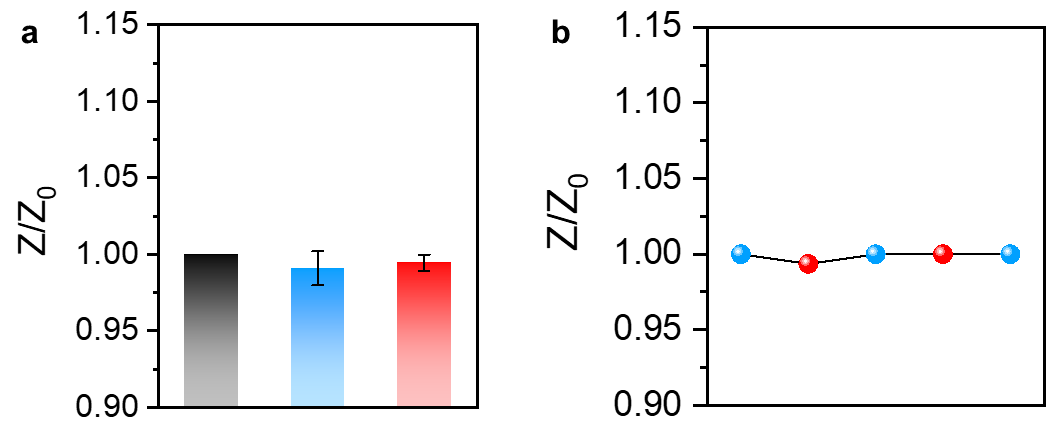


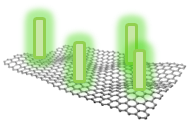


≡


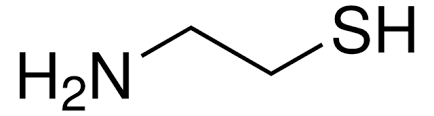


**Fig. S5.** Changes in impedance modulus ratio (*viz. Z/Z_0_*) and cyclability of impedance modulus switch as a function of the magnetic field direction achieved for a-GeCys, where a-Cys is cysteamine. Inset: chemical formula of a-Cys.


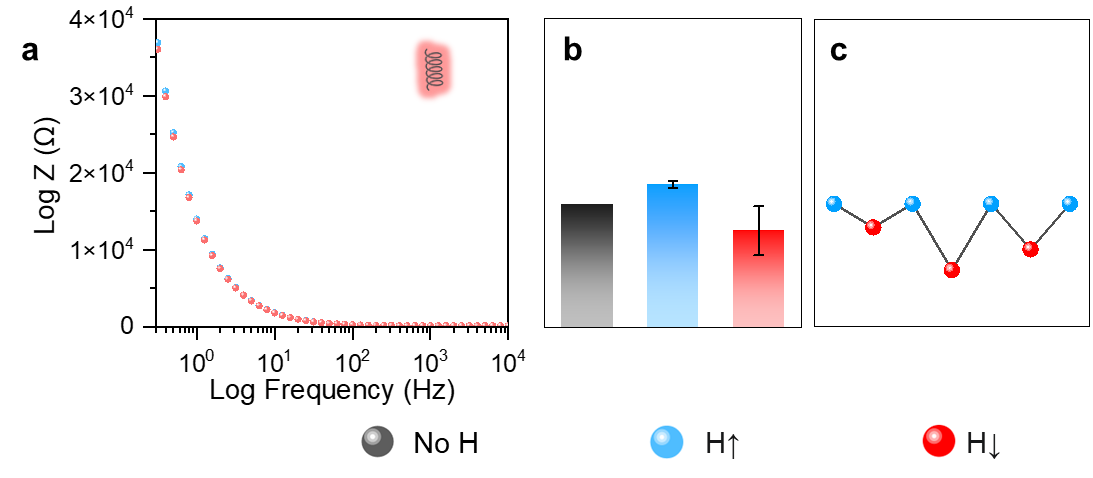


**Fig. S6. (a)** Bode plots of Log Z *vs.* Log Frequency (Hz), **(b)** Changes in impedance modulus ratio (*viz. Z/Z_0_*), and **(c)** cyclability of impedance modulus switch as a function of the magnetic field direction achieved for isolated L-Cys, showing the changes in *Z* after magnetization with both north (H↑) and south (H↓) poles. Electrolyte: 10 mM PBS buffered solution (pH = 7.2). Voltage: 0.1 V; Frequency: 10 kHz to 0.1 Hz.


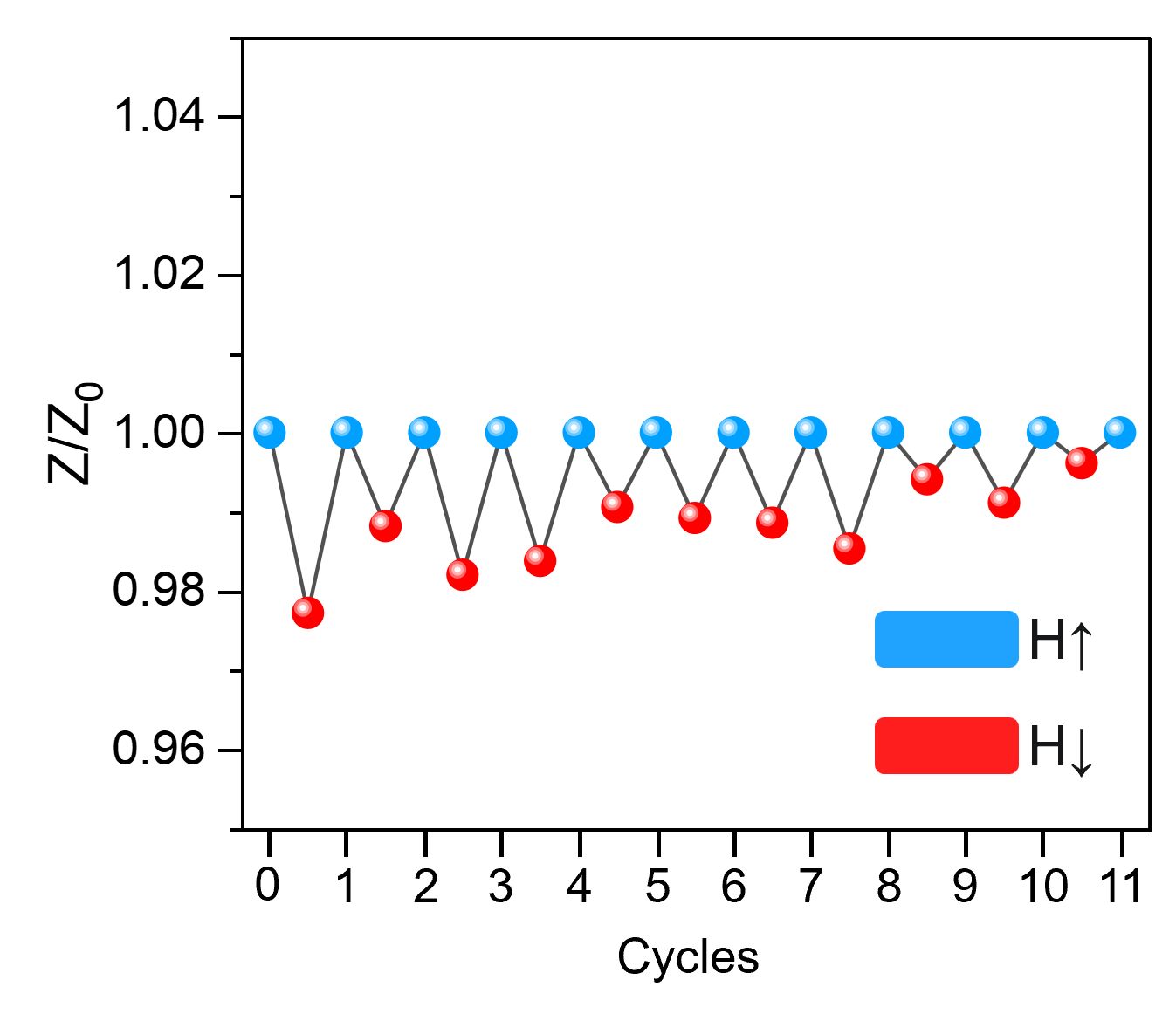


**Fig. S7.** Multi-cycle stability test for L-GeCys.


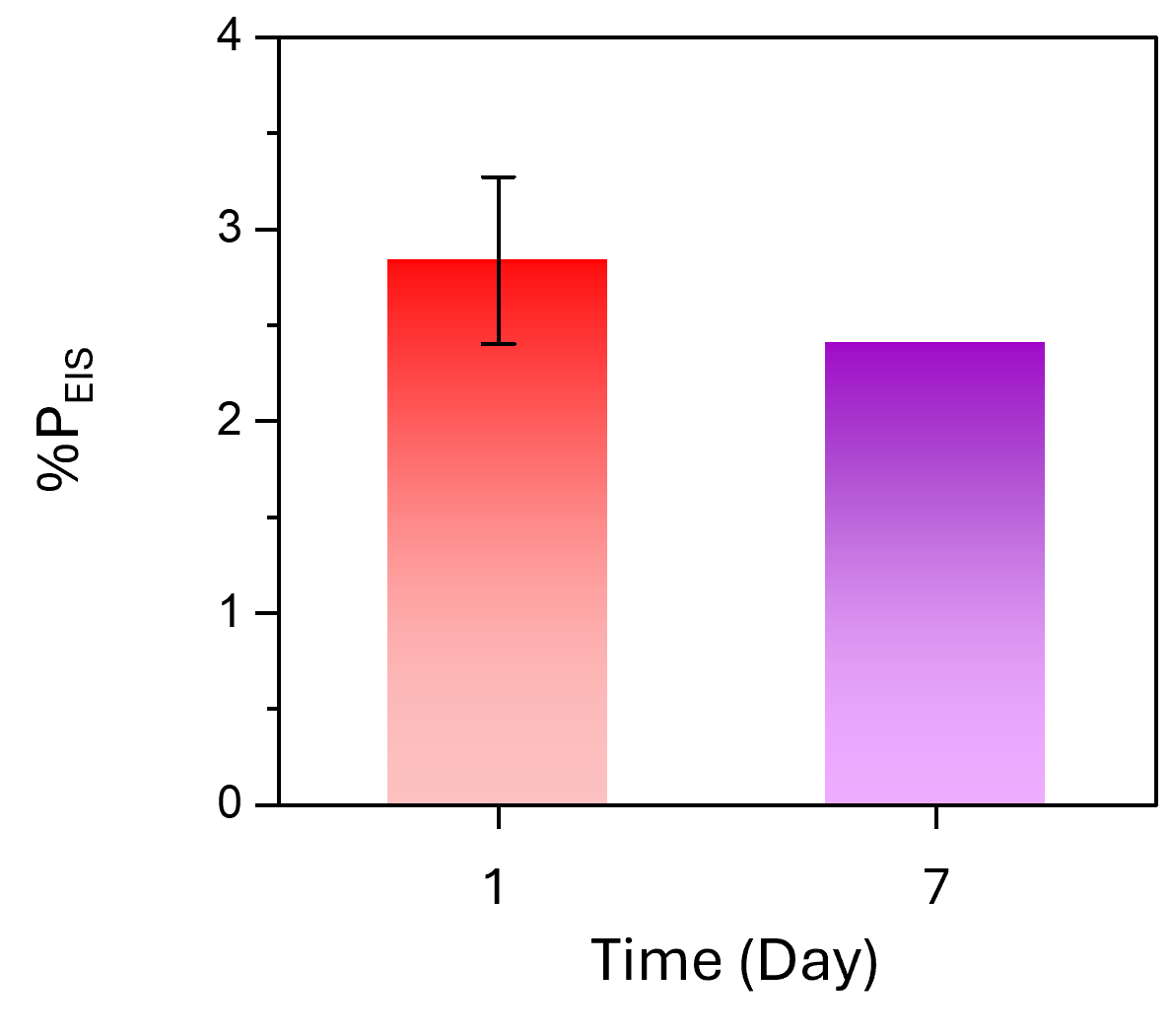


**Fig. S8.** Short-term stability test for L-GeCys after 1 and 7 days.


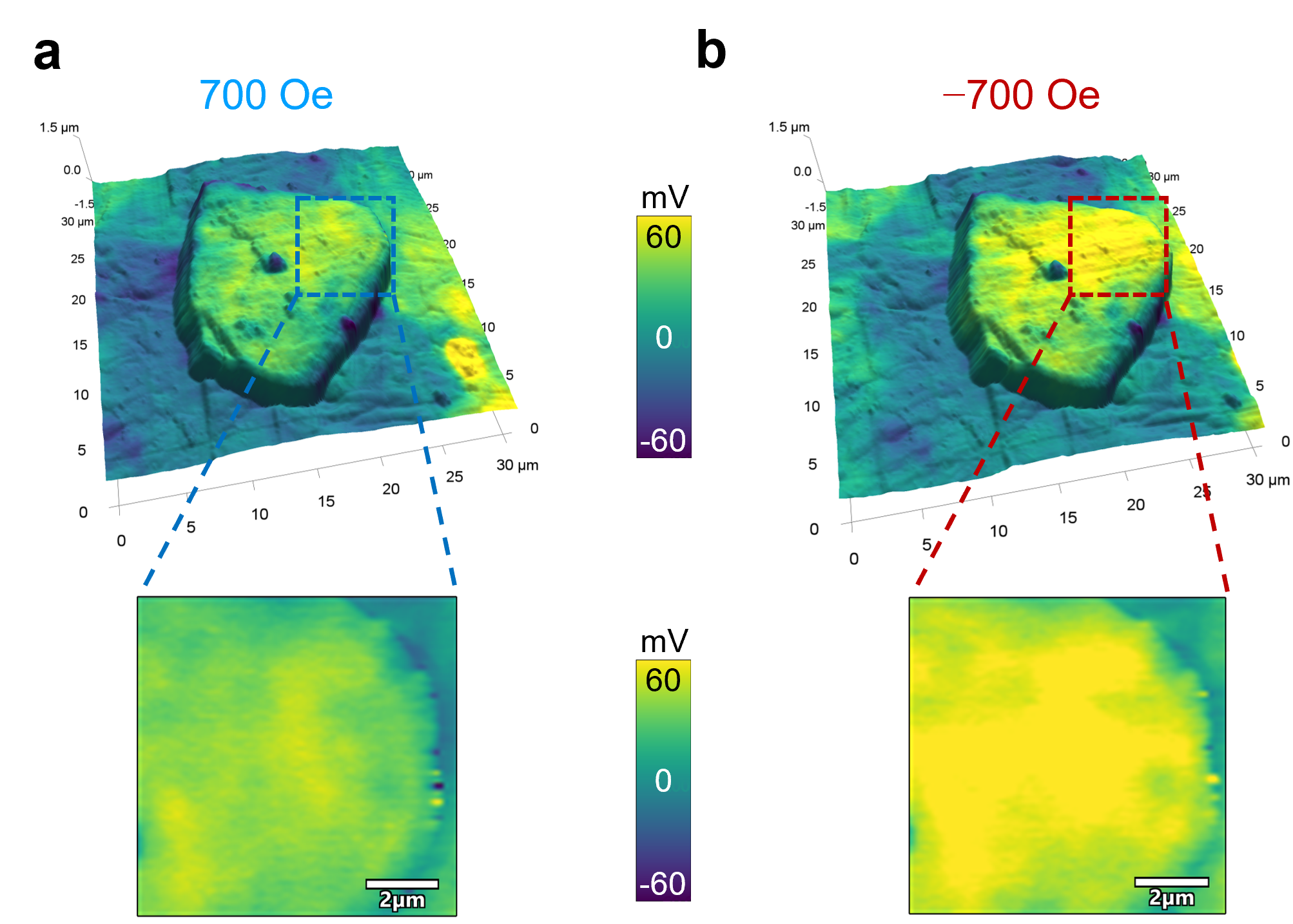


**Fig. S9** KPFM images and surface potentials of L–GeCys drop-casted on ferromagnetic Au@Ni substrate under the magnetic field intensities of **(a)** +700 and **(b)** –700 Oe.


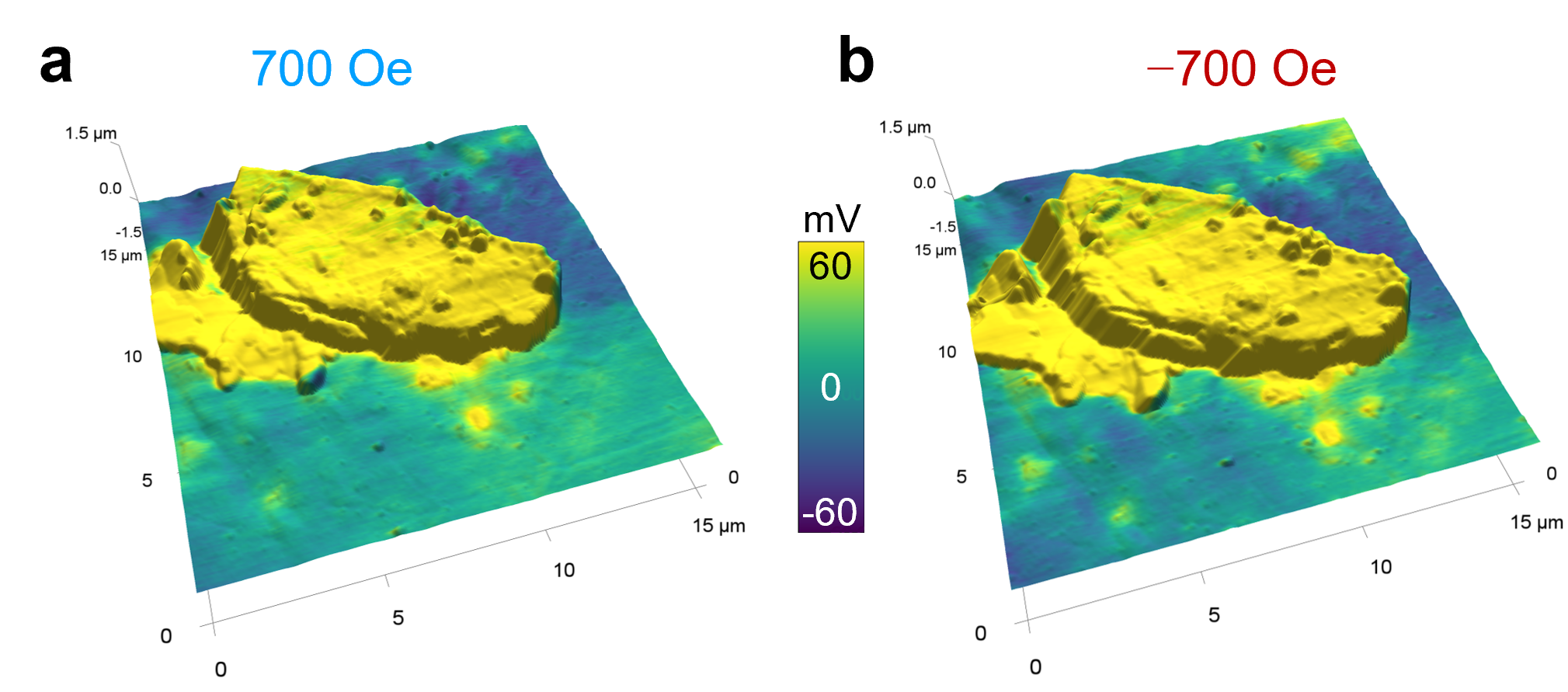


**Fig. S10** KPFM images and surface potentials of 2D–GeH drop-casted on ferromagnetic Au@Ni substrate under the magnetic field intensities of **(a)** +700 and **(b)** –700 Oe.

**Supporting Table**

**Table S1.** Auc values and percentage distribution of chemical bonds for L-GeCys according to its corresponding high-resolution XPS spectra of Ge 3d.

| **L-GeCys** | **Ge–Ge** | **Ge–H** | **Ge–O** | **Ge–S** |
| --- | --- | --- | --- | --- |
| **Auc** | 2101.8 | 973.7 | 294.3 | 1953.4 |
| **%Distribution** | 39.5% | 18.2% | 5.5% | 36.7% |
